# Multilevel perceptions of the virtual delivery of the University of California Diabetes Prevention Program on RE-AIM domains due to COVID-19 mandates

**DOI:** 10.3389/fpubh.2024.1327429

**Published:** 2024-03-08

**Authors:** Tamra Burns Loeb, Maryam Gholami, Kate Ramm, Kelly Shedd, Samantha Soetenga, Nicholas J. Jackson, Un Young Rebecca Chung, O. Kenrik Duru, Carol M. Mangione, Alison B. Hamilton, Tannaz Moin

**Affiliations:** ^1^Department of Psychiatry and Biobehavioral Sciences, David Geffen School of Medicine at the University of California, Los Angeles, Los Angeles, CA, United States; ^2^Altman Clinical and Translational Research Institute (ACTRI), University of California, San Diego, San Diego, CA, United States; ^3^UCI Health, University of California, Irvine, Irvine, CA, United States; ^4^UCLA Campus Recreation, University of California, Los Angeles, Los Angeles, CA, United States; ^5^VA Greater Los Angeles Healthcare System, Los Angeles, CA, United States

**Keywords:** Diabetes Prevention Program, University of California, virtual delivery, multilevel constituents, RE-AIM

## Abstract

**Background:**

The University of California’s Diabetes Prevention Program (UC DPP) Initiative was implemented across all 10 UC campuses in 2018. The COVID-19 pandemic and accompanying mandates required swift changes to program delivery, including pivoting from in-person to virtual delivery (i.e., Zoom). Our goal was to assess multilevel constituent perceptions of the use of a virtual platform to deliver UC DPP due to COVID-19 mandates.

**Methods:**

We conducted qualitative interviews with 68 UC DPP participants, coordinators, and leaders to examine the use of virtual platform delivery on the reach, effectiveness, adoption, implementation, and maintenance (RE-AIM) of UC DPP. Transcripts were analyzed using rapid qualitative analysis and emergent themes were categorized using domains corresponding to RE-AIM framework.

**Results:**

Among UC DPP *participants* (*n* = 42), virtual delivery primarily impacted perceptions of UC DPP effectiveness and implementation. Some participants perceived program effectiveness to be negatively impacted, given their preference for in-person sessions, which they felt provided more engagement, peer support, and accountability. Implementation challenges included problems with virtual format (e.g., “Zoom fatigue”); however, several benefits were also noted (e.g., increased flexibility, maintenance of DPP connections during campus closures). UC DPP *coordinators* (*n* = 18) perceived virtual delivery as positively impacting UC DPP reach, since virtual platforms provided access for some who could not participate in-person, and negatively impacting effectiveness due to reduced engagement and lower peer support. UC *leaders* (*n* = 8) perceived that use of the virtual format had a positive impact on reach (e.g., increased availability, accessibility) and negatively impacted effectiveness (e.g., less intensive interactions on a virtual platform). Across constituent levels, the use of a virtual platform had little to no impact on perceptions of adoption and maintenance of UC DPP.

**Conclusion:**

Perceptions of the reach, effectiveness, and implementation of UC DPP using a virtual platform varied across constituents, although all groups noted a potential negative impact on overall program effectiveness. Unanticipated program adaptations, including virtual delivery, present potential benefits as well as perceived drawbacks, primarily across the effectiveness domain. Understanding differential constituent perceptions of the impact of virtual delivery can help maximize RE-AIM and inform future UC DPP delivery strategies.

## Background

Prediabetes affects 38% of U.S. adults and increases risk of incident type 2 diabetes, a leading cause of morbidity, mortality, and healthcare costs in the U.S. (1, 2). Approximately 1 in 3 U.S. adults had prediabetes in 2019, and without intervention, a significant number are projected to develop incident type 2 diabetes within 5 years (2). The Diabetes Prevention Program (DPP) is a year-long intensive lifestyle intervention which has demonstrated efficacy to lower type 2 diabetes risk among at-risk individuals and those diagnosed with prediabetes (2, 3).

In 2018, the University of California (UC) implemented the DPP across all 10 UC campuses to augment obesity and diabetes prevention efforts, primarily aimed at employees. The UC DPP intensive lifestyle intervention adheres to and is certified by the Centers for Disease Control and National Diabetes Prevention Program (2, 4). The UC Diabetes Prevention Program (UC DPP) is offered free of charge to all UC faculty and staff at risk of developing type 2 diabetes as well as those diagnosed with prediabetes (as defined by the CDC National DPP criteria). The primary outcome of interest for the UC DPP trial was mean percent weight change at 12-month follow-up; secondary outcomes included mean percent weight change at 24-month follow up, challenges and facilitators associated with implementation, and degree of program adoption and maintenance [see (4)]. The evaluation of the UC DPP included diverse UC data sources, including electronic health record (HER) data, administrative claims, campus-based DPP cohort data, site visits, and qualitative interviews (the data source analyzed in the current study) [see (4)]. Our decision to use of qualitative interviews is consistent with the continued need for research utilizing and reporting applications of RE-AIM (5).

In 2020, the COVID-19 pandemic-related public health mandates necessitated unplanned, immediate changes to program delivery, including transitioning from in-person to virtual delivery. All 10 campuses shifted to virtual delivery using the UC Zoom platform due to mandated campus closures. This abrupt shift in program delivery was necessary to continue to offer UC DPP to participants at risk for developing type 2 diabetes and mitigate progression to diabetes among those diagnosed with prediabetes. Research suggests that approximately half of Americans gained weight during the pandemic; this risk was more pronounced among those who reported being overweight before the pandemic (6). Although UC DPP continued to be offered utilizing a virtual platform, there is a lack information about how this change in delivery differentially impacted the perceptions and experiences of UC DPP participants, coordinators, and leaders across RE-AIM domains.

Research comparing in-person to virtual DPP delivery suggests that intensive, multifaceted online DPP programs may be as effective as in-person DPP (7). Offering DPP online can also expand reach to at-risk individuals, although barriers (e.g., lack of internet access, technology, slow internet speed, lack of quiet space) have been noted (8). However, despite the CDC Diabetes Prevention Recognition Program Registry’s recognition of online delivery of DPP (9), there are gaps in our knowledge about perceptions of the virtual delivery of preventative health care programs. Accordingly, the purpose of this study is to assess multilevel perceptions of delivering the UC DPP Initiative virtually due to the COVID-19 pandemic utilizing the RE-AIM (reach, effectiveness, adoption, implementation, and maintenance) framework (10, 11).

## Methods

A planned component of the overall evaluation of the UC DPP was the use of both quantitative and qualitative data to maximize the use of the RE-AIM model (5) In-depth qualitative interviews, an ideal method to better understand multilevel stakeholder perceptions, experiences, and opinions with respect to the UC DPP, were conducted (4, 12). To accomplish this goal, the RE-AIM framework guided the development of a semi-structured interview guide (5), with versions tailored to DPP participants, coordinators, and leaders. Participants were individuals that participated in UC DPP sessions. Coordinators helped to support the UC DPP at one site, and Leaders were those that provided support across sites; many were affiliated with the University of California, Office of the President.

We sent study invitation emails and letters to UC key constituents between February and July 2021. In-depth qualitative interviews were scheduled with interested constituents. Interviews were conducted by a trained qualitative team member over UC Zoom and lasted approximately 1 h. Participants included those that engaged in UC DPP entirely in-person (pre-pandemic), virtually (during the pandemic), and a combination of in-person and virtually (those that transitioned to virtual from in-person at the beginning of the pandemic, when stay-at-home mandates were imposed).

Three interview guides informed by the RE-AIM framework were developed for the following partner groups: (1) participants, (2) coordinators, and (3) leaders. Participant interview guides assessed RE-AIM domains, including Reach (i.e., whether they had received a diagnosis of prediabetes, number of sessions attended), Effectiveness (i.e., what parts of the program worked and did not work for them, their overall satisfaction, suggestions for improvement, whether they participated in DPP on Zoom because of COVID-19 stay-at-home restrictions, and if so, how they would rate the virtual experience and how it could compare to an in-person option), Adoption (i.e., whether UC DPP affected their health and wellness, level of physical activity, eating habits, stress level, and emotional and social health, whether the sessions helped them meet the goals they set for themselves, and how the program helped with accountability), Implementation (i.e., specific program components, including the enrollment process, materials, their group coach, group interactions, and timing and frequency of sessions, as well as facilitators and challenges to participation), and Maintenance (i.e., whether they continued any lifestyle changes they made in the program and whether the program should be offered in-person, virtually, or both). Participants also responded to a question about how COVID-19 impacted participation in UC DPP.

Coordinator and leader guides were similar; coordinators were asked about their specific campus and leaders responded to questions across the UC system. Consistent with the participant guide, coordinator and leader guides also assessed RE-AIM domains, including Reach (i.e., strategies used to raise awareness of UC DPP and outreach/recruitment strategies), Effectiveness (i.e., how they evaluate UC DPP effectiveness, strengths, areas in need of improvement, feedback collected, and specific program components, including materials, training sessions, coaches, and data collection), Adoption (i.e., facilitators and barriers), Implementation (i.e., adaptations made to the program to meet campus or participant needs), and Maintenance (i.e., program needs and obstacles to sustainment). Coordinators and leaders also responded to several questions about the COVID-19 pandemic (i.e., how COVID-19 impacted UC DPP) (see Table 1 for a summary of constituent interview guides).

**Table 1:** UC DPP participant, coordinator, and leader interview guides.

RE-AIM domain	Participant interview guide	Coordinator and leader interview guide
Reach	Responded to questions about whether they had prediabetes, and if so, who told them, and how the diagnosis made them feel and changed how they thought about their health. They also reported the number of sessions attended.	Asked about strategies used to raise awareness of UC DPP, outreach/recruitment strategies, whether participants reflect the campus population, efforts to ensure a diverse representation of at-risk individuals, how to increase reach, and thoughts about why some faculty and staff participate or decline participation in UC DPP.
Effectiveness	Described what parts of the program worked and did not work for them, their overall satisfaction, suggestions for improvement, whether they participated in virtual DPP on Zoom due to COVID-19 restrictions, and if so, how they would rate the virtual experience and how it would compare to an in-person option.	Asked questions about how they evaluate UC DPP, as well as the effectiveness, strengths, areas in need of improvement, feedback collected, and specific program components (i.e., materials, training sessions, coaches, and data collection).
Adoption	Asked about whether UC DPP affected their health and wellness, level of physical activity, eating habits, stress level, and emotional and social health. Participants recounted the goals they set for themselves, whether it became easier to meet their goals as they attended more sessions, whether the sessions helped them meet their goals, if the DPP recommended goals were attainable, and how the program helped with accountability.	Asked about facilitators and barriers to UC DPP adoption and ways the UC community and participants benefitted from the program.
Implementation	Asked about specific program components, including the enrollment process, materials, their group coach, sessions, group interactions, and timing and frequency of sessions. They also were asked what made it easier to participate, challenges to participation, and whether they had to make changes to participate in the sessions.	Implementation questions focused on adaptations made to the program to meet campus or participant needs, and any unintended consequences of the program.
Maintenance	Asked if they continued any lifestyle changes, they made in the program and whether they thought the program should be offered in-person, virtually, or both.	Questions included program needs and obstacles to sustaining UC DPP.
COVID-19 pandemic	Asked if and how COVID-19 impacted participation in UC DPP.	Asked about how they balanced involvement with UC DPP with campus priorities during the pandemic; how COVID-19 impacted UC DPP.

Coordinators and Leaders answered similar questions; Coordinators were asked about their specific campus and Leaders responded across the UC system. Coordinators were asked to provide their opinion about specific program components.

### Qualitative data collection

Interviews were recorded, professionally transcribed, and transcripts were reviewed in detail by the research team. After familiarization with the transcripts, the team used rapid qualitative analysis, a type of manifest content analysis developed for and utilized in health services and implementation research, for example to aid in the rapid identification or expansion of knowledge of intervention components as well as facilitators and barriers of a program, to analyze the data (13, 14). Constituent (i.e., participant, coordinator, and leader) responses that alluded to delivery and use of the virtual platform with respect to RE-AIM domains were reviewed and synthesized. A templated summary of each transcript was created, creating a multilevel inventory of constituent responses to each of the respective interview guide domains. These summaries were combined into matrices to identify and compare themes, as well as to establish thematic saturation [i.e., sufficient, cross-cutting evidence for the multilevel themes presented below; (15)] related to the use of the virtual platform (13). The study was approved by the UCLA Institutional Review Board. All constituents provided verbal consent and were offered a $50 gift card incentive after the interview was completed.

## Results

Between April and August 2021, 68 constituents (42 UC DPP participants, 18 coordinators, and 8 leaders) completed interviews. The UC DPP participants’ mean age was 46 years (9.8); 33 (79%) were female and 9 (21%) were male. Thirteen (31%) identified as Asian, 8 (19%) Caucasian, 12 (29%) Latino, 1 (2%) Black, 2 (5%) American Indian/Alaskan, or Native Hawaiian/Pacific Islander. Six participants (14%) did not report their racial/ethnic background. Three participants (7%) reported receiving some college education, 27 (64%) had a college degree, and 12 (29%) had an advanced degree. UC DPP coordinators and leaders did not report demographic characteristics for this study.

### UC DPP participants

UC DPP participants perceived virtual delivery as having the greatest impact on effectiveness and implementation domains. Few participants commented on reach, but several noted that other group members had dropped out because of the shift to a virtual platform, and one said they were not provided with a virtual option. The majority of participants perceived program *effectiveness* to be negatively impacted by virtual delivery, given their preference for in-person sessions, which provided more engagement and accountability,

“…I feel like [the virtual option] is not as engaging, if that makes sense. I feel like people are there, but they’re not really…there. Like you talk but most of the time, everybody kept their cameras off and sometimes it was just like, anyone there, have any questions or suggestions, you know?”

Another participant echoed this sentiment, stating,

“I think what was missing…what’s missing from the virtual, I think, is that like I said, that in-person camaraderie, the motivation, like I say, weighing in together, just physically being in the room with somebody, you know, it’s expressive. There is a lot of thought and emotion tied to this subject. So, I think just that compassion for just having that in- person experience. Over Zoom you can’t really gauge someone’s…I don’t know, facial expressions are everything. I think it just enhances the experience.”

Others noted, “So over remote, it was a lot of quiet and everyone mostly—a lot of the time, we all had our cameras off versus being able to see a person,” and “The Zoom was just – you are distracted more easily, you know?” Some described reduced interaction with group members and the facilitator, and one stated that it was more difficult to get feedback. Participants also described in-person delivery as easier, presenting fewer obstacles, and included seeing and interacting with others. According to one participant,

“I think once we went remote and obviously, we never went back—I mean we’re still not back yet I think—I can’t really say because I don’t think it’s fair to say what didn’t work because I don’t think it went as anybody planned. I think it worked as best it could in the remote environment. But I don’t have anything to compare it to because what was presented in person, I believe would have been super beneficial. Like I said with the weight bands, with the portion plates, it would have been maybe more interactive. We all weighed in together, so I think that part could have really been even more motivational, inspirational, coming together in that sense.”

In contrast, several participants noted that virtual was as effective as in-person programming. Participants indicated that “we actually did fine with it,” “It was good, and that they did not believe it made a “tremendous difference in the nature of the program.” Another participant noted,

“I think it’s a great program, to be honest. I’m excited—if they were to offer it and if I could potentially participate again and if it works with my schedule, I would definitely do it… I think it’s great. I think it lost some momentum with the pandemic, but yeah, I really enjoyed the program for the most part.”

Another stated,

“It was fine. We didn’t have connectivity issues. We went the whole time. I didn’t feel like it was anyhow shortened or less informative or we were missing something because we really still kept talking. I think the group had a lot of commitment to finish this program for the whole year and go through it and continue learning as much, because there was always—there was never a session that did not have a question, a suggestion, or sharing a tip. You could count on that every meeting, so it showed that the other participants were equally invested and involved. It wasn’t like it was just one person. Because you know sometimes when you’re in Zoom, it’s either just one person or it’s dead silent or it’s hard. That did not happen in these meetings.”

With respect to *adoption*, participants noted that DPP was no longer a priority or took a back seat to other pandemic-related concerns, and that they did not own and had to purchase a scale for home use to continue the program. One participant described the shift to virtual having no impact on adoption. With regard to *implementation*, participants’ comments focused on implementation challenges, including problems with virtual format (e.g., “Zoom fatigue” or having to choose between taking a lunch break or participating in the Zoom session). One participant noted, “It was hard to do the virtual after kind of 10 h of non-stop virtual for work;” another stated, I “really did not like the Zoom meeting because my full day was Zoom meetings and I really just kind of let that go.” Others described scheduling challenges, being restricted to exercising at home, competing demands (i.e., children at home), technical issues or having to learn how to use Zoom, having to weigh oneself, and having to be honest about lifestyle self-management from home. One participant stated,

“COVID just shut everything down. We went remote. We didn’t receive the materials we were supposed to receive. We didn’t receive—we were supposed to get portion plates and I think something else. And you know, it was out of everybody’s control, but we still kept meeting.”

Others noted that the virtual format was easier or preferred: “I think, yeah, probably the Zoom did make it easier. I think if we were in person on campus…. We would have to walk there or find our way there in person. And so with the Zoom, we just log in and there we are.” Another stated, “It (virtual delivery) was good. Yeah. Yeah, it was good. I mean, it worked. It worked out.” Some participants noted that the virtual platform helped them to stay connected with others during campus closures. While participants were asked questions about maintenance, they did not describe the impact of virtual delivery on this dimension (see Table 2).

**Table 2:** Multilevel constituent perceptions of use of a virtual platform to deliver UC DPP across RE-AIM domains.

RE-AIM Domains	Perceptions (+, −)	Participants (*n* = 42)	Coordinators (*n* = 18)	Leaders (*n* = 8)
Reach	+		Increased retention	Increased Availability Accessibility
	−	Increased attrition	Increased attrition	
Effectiveness	+			
	−	Reduced: Interactions with group members and facilitator Accountability Visibility Feedback Increased: Distractions Obstacles	Reduced: Momentum Peer support Accountability Engagement Increased: Awkwardness	Reduced: Interaction among participants Visibility Increased: Interruptions
Adoption	+			
	−	Competing demands during pandemic Had to purchase scale	Competing demands during pandemic	Reduced participants’ effort to be visible
Implementation	+	Easier than in-person Helped to stay connected with others		
	−	Problems with: Zoom fatigue Scheduling challenges Competing demands Technical issues Low digital literacy Accountability	Problems with: Zoom fatigue Feeling limited in what UC DPP could provide Difficulties collecting participant data	
Maintenance	+			
	−			Concerns about lack of long-term funding

### UC DPP coordinators

UC DPP coordinators described the virtual delivery having the greatest impact on the reach and effectiveness domains. Some noted that the virtual platform increased *reach* among those who could not participate in-person: “Retention has been much better in the virtual world.” Others stated that participants were lost due to the virtual transition, and that it had no or a similar effect. They also described virtual platforms negatively impacting *effectiveness* due to Zoom being awkward or less engaging, loss of momentum, decreased peer support, and reduced accountability. Similar to participants, coordinators also described DPP as less of a priority in the context of the pandemic, negatively affecting *adoption*. With respect to *implementation*, coordinators discussed negative impacts, including Zoom fatigue, feeling limited in what the program could provide, and difficulties collecting participant data. One noted,

“Some campuses—again in just this COVID world, they are having a harder time reaching people. So that’s been the biggest struggles. And then engagement in this virtual world, in the beginning it was more novel and exciting. They’re like, ‘Oh, I can still see you and it’s Zoom’ and…we can still do this. And I think people still like it but they are kind of over it, as well…I think it does work really well for some people, but again, it’s finding what works for everyone. Having an option for an in-person and a virtual, depending on what meets the need.”

While coordinators were asked questions pertaining to *maintenance*, they did not describe any effects of virtual delivery on this dimension.

### UC DPP leaders

Leaders described several benefits of virtual delivery on *reach*, including increased availability and accessibility. One leader noted,

“I think the teams have been phenomenal in transitioning from in-person to remote meetings… Funding has been especially challenging in the COVID environment, and I also think that the program loses visibility when folks aren’t on campus. They’re not talking to each—they’re not having hallway conversations with others. I do think it’s presented challenges, but I think the team has adjusted phenomenally.”

Drawbacks were noted for *effectiveness*, including reduced interaction among UC DPP participants on the virtual platform, less visibility, and interruptions (i.e., starting and stopping). One leader stated,

“A lot of the value of this program is the people getting to know each other and providing support for each other that are participating in the program every week. And you can continue to do that on the Zoom format, but you’re not going to create the same kind of personal bonds that you would in person where there’s a lot of chatter before and after meetings and stuff like that. I don’t think it’s the same…. But it’s still better than not having the program at all.”

With respect to *adoption*, one leader noted that there was a decreased effort to be visible. Leaders did not comment on *implementation*. One leader expressed *maintenance* concerns related to lack of secure funding.

## Discussion

This study identified multilevel perceptions of the virtual delivery of a CDC-recognized lifestyle behavior change program, the UC DPP, across all 10 UC campuses. In 2018, the UC system, the third largest employer in California, prioritized diabetes prevention as a system-wide goal, offering a worksite behavior change program, the UC DPP, free of charge to all UC employees with documented prediabetes or who are at risk of developing type 2 diabetes. UC DPP groups are led by UC staff who have completed DPP coach training and are experienced in delivering campus-based wellness programs (4).

Despite significant documented increases in telemedicine (e.g., the provision of clinical services) and telehealth (e.g., health-related services, including administration and continuing medical education) during the pandemic, as well as research focused on patient satisfaction with these services (16), far less is known about multilevel constituents’ perceptions surrounding the shift of lifestyle behavior change programs to virtual delivery. Our study found that perceptions of virtual delivery on RE-AIM domains of UC DPP varied across constituent groups, with most reporting a negative impact of virtual delivery on program effectiveness. This study provides evidence that unanticipated program adaptations, including shifting to virtual delivery, present potential benefits as well as perceived drawbacks across RE-AIM domains.

UC DPP participants reported negative effects of virtual delivery across reach, effectiveness, adoption, and implementation domains, with some indicating that virtual delivery had effectiveness and implementation benefits. Future research should focus on facilitating program effectiveness, participant engagement, accountability, interaction, and providing feedback using virtual DPP. The impact of virtual delivery due to COVID-19 on maintenance was limited in this study as it was conducted mid-pandemic; future research should focus on understanding the effects of virtual UC DPP delivery on the maintenance dimension. Barriers described by participants included Zoom fatigue, scheduling, and technical challenges, and competing demands. For participants who face these challenges, in-person DPP delivery may be preferable. Reducing these barriers should increase perceived effectiveness and implementation among other participants.

UC DPP coordinators discussed negative consequences of virtual delivery across reach, effectiveness, adoption, and implementation domains. There were equal numbers of remarks about positive (or neutral) and negative effects of virtual delivery on reach. Coordinators did not perceive other positive benefits to virtual delivery. While UC DPP leaders also discussed the drawbacks of virtual delivery on effectiveness and adoption, they described positive impacts on reach. Research designed to leverage the benefits of UC DPP delivery using virtual platforms and mitigate barriers from the participant, coordinator, and leader perspectives is needed. Understanding the differential impact of these pandemic-related changes can help maximize RE-AIM and inform future strategies for UC DPP delivery.

Limitations of the current study include the inability of each constituent group to plan and prepare for the shift to virtual delivery and to fully anticipate barriers and facilitators to engagement, due to the sudden onset of COVID-19 and accompanying stay at home mandates. Although the abrupt shift to virtual delivery allowed for continuity of UC DPP programming, our understanding of the extent to which socioeconomic factors, lack of technology, and/or low digital literacy affected participants’ ability to engage in the program is limited to the remarks provided by participants in these interviews. These challenges included “Zoom fatigue,” scheduling difficulties, competing demands (including having children at home), an inability to exercise outside of one’s residence, and low digital literacy. Future research should examine the differential impact of these and other contextual factors on participants’ ability to engage in virtual lifestyle change programs. This study sample is comprised entirely of the recollections of UC faculty and staff; the extent to which their perceptions are generalizable to constituents from other institutions of higher education awaits future investigation.

## Conclusion

The UC system prioritized diabetes prevention as a system-wide goal, offering UC DPP free of charge to all UC employees at risk for or diagnosed with prediabetes, levering campus wellness resources in diabetes prevention, and shifting to virtual delivery during the COVID-19 pandemic to maintain program continuity. This study examined perceptions of utilizing a virtual platform (UC Zoom) to deliver UC DPP on RE-AIM domains. Perceptions varied across constituent groups, with most describing a negative impact of virtual delivery on program effectiveness. There is a need to develop questions to assess preferences for and potential barriers to virtual delivery, include them in the data routinely collected for the CDC, and refine strategies for UC DPP implementation accordingly. Given that remote and/or hybrid DPP delivery is likely to continue, identifying and addressing the challenges and opportunities of the virtual delivery of UC DPP across the RE-AIM domains is critical for ongoing diabetes prevention programming efforts.

## Data availability statement

The raw data supporting the conclusions of this article will be made available by the authors, without undue reservation.

## Ethics statement

The studies involving humans were approved by University of California IRB# 20-000357. The studies were conducted in accordance with the local legislation and institutional requirements. The participants provided their written informed consent to participate in this study.

## Author contributions

TL: Writing – review & editing, Writing – original draft, Formal analysis, Data curation. MG: Writing – review & editing. KR: Writing – review & editing, Formal analysis. KS: Writing – review & editing, Formal analysis. SS: Writing – review & editing, Formal analysis. NJ: Writing – review & editing. UC: Writing – review & editing. OD: Writing – review & editing. CM: Writing – review & editing. AH: Writing – review & editing, Formal analysis. TM: Writing – review & editing, Writing – original draft, Methodology, Investigation, Funding acquisition, Formal analysis, Conceptualization.
